# Censoring in Clinical Trials: Review of Survival Analysis Techniques

**DOI:** 10.4103/0970-0218.66859

**Published:** 2010-04

**Authors:** Shankar Prinja, Nidhi Gupta, Ramesh Verma

**Affiliations:** London School of Hygiene and Tropical Medicine, Keppel Street, London, UK; 1 Department of Radiotherapy, Government Medical College, Chandigarh, India; 2 Department of Community Medicine, Pt BDS PGIMS, Rohtak, Haryana, India

## Introduction

Medical and epidemiological studies are mostly conducted with an interest in measuring the occurrence of an outcome event. Studies conducting survival analysis, however, are focussed toward measuring time to event or outcome. Time to event could vary from time to fatal event i.e. death, or time to occurrence of a clinical endpoint such as disease, or attainment of a biochemical marker. Survival analysis studies originated with the publication of John Graunt’s Weekly Bills of Mortality in London. First life table was prepared by Healy.(1) Survival analysis is also known as lifetime data analysis, time to event analysis, reliability and event history analysis depending on focus and stream where it is used. However, survival analysis is plagued by problem of censoring in design of clinical trials which renders routine methods of determination of central tendency redundant in computation of average survival time. The present essay attempts to highlight different methods of survival analysis used to estimate time to event in studies based on individual patient level data in the presence of censoring. Section 2 highlights types of censoring encountered in a clinical trial, its types and potential statistical solutions. Survival analysis techniques, its assumptions and suitability of methods under different data conditions are illustrated in sections 3 and 4. The next section 5 discusses the importance of techniques to extrapolate estimate of life expectancy derived over a period of time exceeding the duration of trial follow-up. Lastly, section 6 cites limitations and advantages of different methods and finally concludes by indicating possible future areas of research and practice for health economists and public health professionals. We reviewed articles published in PubMed, Science Direct and Ovid search engines using “censoring in clinical trials”, “survival analysis” and “Kaplan Meier method” as key words. After a total of 213 articles retrieved, articles focussing only on methodology aspect were considered for the present review. Original articles were preferred following by subsequent discussion articles, which added substantially to the methodology.

## Censoring in Clinical Trials

Censoring is said to be present when information on time to outcome event is not available for all study participants. Participant is said to be censored when information on time to event is not available due to loss to follow-up or non-occurrence of outcome event before the trial end. Broadly classifying two types of censoring are encountered, i.e. point and interval censoring.(2)

**Figure 1:**
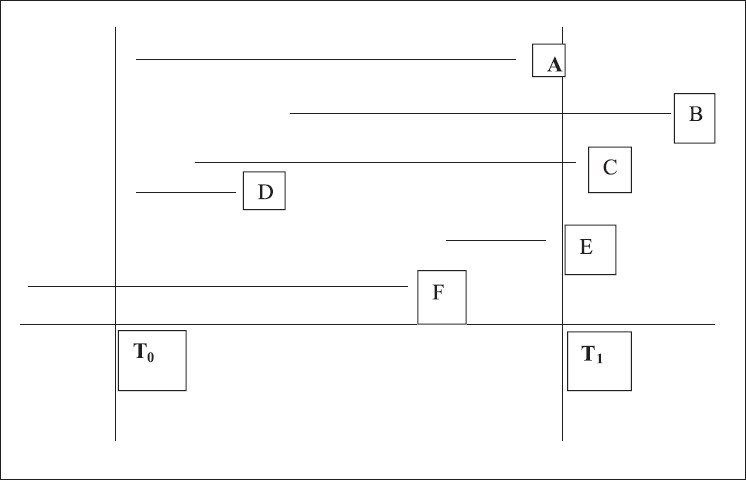
Point censoring in clinical trial

*Point censoring* is said to occur when despite *continuous monitoring* of outcome event, the patient is lost to followup or the event does not occur within the study duration. It is also known as right censoring which can be either end-of-study censoring or loss-to-follow-up censoring. An individual is said to be left censored if the patient had been on risk for disease for a period before entering the study. However, left censoring is usually not a problem in clinical trials, since starting point is defined by an event such as entry of patient in trial, randomization or occurrence of a procedure or treatment. Individuals B and C are right censored while individual F is left censored [Figure 1].

**Figure 2:**
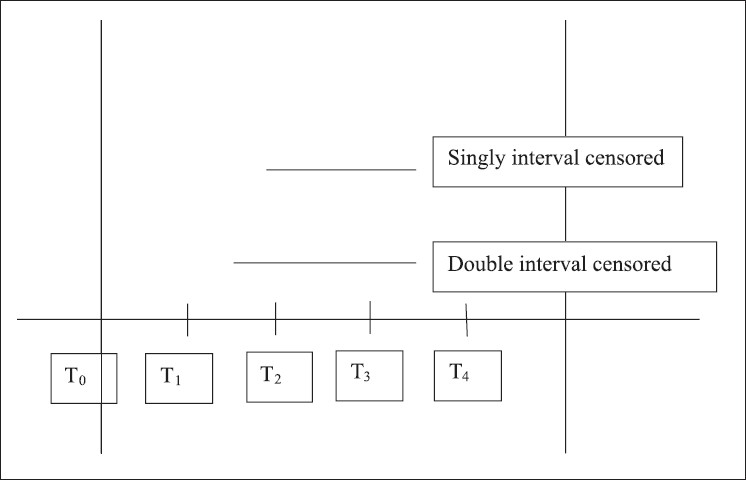
Interval censoring in a clinical trial

Problem of interval censoring arises when time to event may be known only up to a time interval. This situation occurs in case the assessment of monitoring is done at a periodical frequency. This is illustrated by a hypothetical study done to ascertain the incubation period of AIDS after occurrence of HIV infection [Figure 2]. Practically, most observational studies dealing with non-lethal outcomes have periodical examination schedules and are thus interval censored. However, if the periodicity of examination is at a justified frequency, interval censored data can be dealt with as point censored.(3)

Statisticians have devised various methods to deal with censored data which includes complete data analysis, imputation techniques or analysis based on dichotomized data.(2) However, these methods are laden with problems and complexities for others. Detailed discussion of each of these methods is beyond the scope of the present essay due to space constraint; however, it is important to bear in mind the techniques available. The more effective methods that are widely used in survival studies encountering censored data are likelihood-based approaches (survival analysis methods) which adjust for the occurrence of censoring in each observation, and thus are advantageous that it uses all available information.

## Survival Analysis Techniques

Survival analysis techniques used for dealing with censored data can be broadly classified into nonparamteric (Kaplan Meier product limit method), parametric (Weibull and exponential methods) and semi-paramteric method (Cox-proportional hazards method). The latter two can also be applied as regression-based models. However, usual likelihoodbased functions of whole sample which are product of individual likelihood functions cannot be applied in the presence of complex censoring mechanisms, especially in the presence of both loss to follow-up and end of study censoring. In such situation, a joint distribution of survival and censoring times can be done. However, the distribution of survival time in such situation is considered as non-identifiable. Hence, the assumption of *independent censoring* is imperative.(2) This assumption implies that time to censoring and survival times are independent. In other words, censoring is independent of unusual high or low risk for occurrence of event which implies that survival times for censored and uncensored individuals is same and removal of censored individuals from analysis would yield an unbiased estimate of survival time or time to event.

## Non-Parametric Survival Analysis: Kaplan Meier Product Limit Method

This method computes the probability of dying at a certain point of time conditional to the survival up to that point.(4) It utilizes the information of censored individuals till the point when the patient is censored. Thus it maximizes utilization of available information on time to event of the study sample. The equation used to derive survival probability at time ‘t’ is derived by the following equation:

$$S\left(t\right)=\;\pi_{t\;}\left(1-d_{t}/n_{t}\right),$$

where d_t_/ n_t_ represents the probability of dying at time ‘t’ conditional to being at risk (alive) at ‘t-1’ time.

The censored individuals are excluded from the denominator of ‘at risk’ individuals at the point when they are censored, however, are included at each preceding point. They are not included in numerator at any point. Tables 1 and 2 illustrate a hypothetical dataset and computation of survival probability using the Kaplan Meier estimator, respectively.

**Table 1:** Hypothetical data to illustrate survival analysis

Patient ID (i)	Time to event (death) or censoring (X_i_)	Censoring indicator variable (1: dead; 0: censored) (δ_i_)
1	7.8	1
2	6.3	1
3	2.7	0
4	4.6	1
5	9.3	0
6	10	1
N	8.2	0

**Table 2:** Hypothetical illustration of estimating probability of survival S(*t_i_*) using Kaplan Meier estimator

*t_i_*	*n_i_*	*d_i_*	1-*d_i_/n_i_*	S(*t_i_*)
2.5	21	3	0.8571	0.8571
4.6	17	1	0.9412	0.8067
5.1	15	1	0.9333	0.7529
5.8	12	1	0.9167	0.6902
6.5	11	1	0.9091	0.6275
7.3	7	1	0.8571	0.5378
10	6	1	0.8333	0.4482

Censoring affects the shape of survival curve in a situation when a large number of individuals are censored at a single point of time leading to sudden spurious large jumps or large flat section in survival curve.(5) Other factors leading to such spurious jump includes extremely low number of individuals at risk especially toward the end of the study or pre-arranged clinic visit schedule. Statistical significance of difference between two or more survival curves is determined by the log-rank test.

Reliability of the different portions of survival curve is dependent on the number of individuals at risk at that stage. Majority of studies dealing with analysis of survival time are likely to have some individuals for which outcome event and thus time to event or outcome is not recorded. This is in view of paucity of resources (money and time) to carry forward the study till outcomes (especially fatal outcomes) are recorded for each and every study individual. To have a measure of maturity of data thus becomes imperative to show quality of data in terms of adequacy of individuals for which outcome event and thus time to event is recorded. Simpler measures for maturity include average (median) follow-up period. However, a more robust graphical technique involves constructing a survival curve by reversing censoring i.e. labelling patients died as censored and vice-versa and computing time to follow-up for this dataset. Thus median time to follow-up is estimated using this technique which deals with censoring in the same manner as for time to survival.(5)

## Parametric Survival Analysis: Weibull and Exponential Methods

As in the non-parametric approaches in the analysis of time to event data, the models under parametric approach derive estimates of failure time statistics while accounting for the presence of censoring in the data. The main difference between the two approaches is that the latter attempt to derive estimates using a parametric model, which male specific assumptions about the distribution of failure time through assuming a particular functional form for the hazard rate. This functional form can specify the hazard rate as a function of time or can incorporate covariate information in which case the hazard rate is specified as a function of time and specific covariates. In this way, failure time is related to a set of covariates thus leading to a regression approach.

Parametric methods of survival analysis assume distribution of hazard rate as a function of time besides assumption of independent censoring.(5) Hazard rate is the instantaneous probability of dying in next short interval conditional upon having survived till time ‘t’. The expression d_t_ /n _t_ in Kaplan Meier product limit survival equation is hazard rate. The denominator n_t_ includes total follow-up time till time ‘t’ for both individuals who are at-risk and censored. Numerator d_t_ is the number of individuals who die/ experience outcome event during the short time interval after ‘t’. Censoring plays a similar role in the models as in the case of the non-parametric hazard and the condition of independent censoring. The censoring is adjusted for in parametric models by incorporation of hazard rate which uses similar Kaplan Meier estimator. When the model incorporates covariates, the condition of independent censoring is assumed in the presence of covariates.

Weibull method assumes that hazard rate changes (increases or decreases) monotonically with time. Hazard rate and other parameters for Weibull distribution are shown in Table 1. Since hazard rate is time dependent, it increases for all values of *P*>1, decreases for *P*<1 and is constant for *P*=1 [Table 3].

**Table 3:** Parameters in Weibull and Exponential methods of survival analysis

Parameter	Survival analysis method	
	Weibull method	Exponential method
Hazard rate	h(t)=λp(λt)^p-1^	h(t)=λ
Probability density function	f(t)=λp(λt)^p-1^e^-(λt)p^	f(t)=λe^-λp^
Survival function	S(t)= e^-(λt)p^	S(t)= e^-(λt)^

Exponential distribution is a special case of Weibull distribution for which *P* =1, implying that the hazard rate does not vary with time. A constant value of hazard rate leads to a constant probability of dying, unchanged with passage of study duration or time. This highlights the fact that the probability of dying during any given interval is dependent only on the width of time interval, with directly proportional relationship. On the contrary, it is unaffected by the point of beginning and end of time interval.

It is important to ascertain the distribution of hazard rate for any dataset. Since the exponential distribution assumes constant hazard rate, this can be written as;

S(t)= e^-λt^,which can also be written as

Log [S(t)]= -λt or - Log [S(t)]= λt. Taking logarithms on each side,

$$\mathrm{Log}\left\{-\;\mathrm{Log}\;\left[S\left(t\right)\right]\right\}=\;\mathrm{log\lambda}\;+\log\;t$$

This is an equation of straight line, with slope ‘b’ equal to 1. Thus complementary log transformation i.e. Log{- Log [S(t)]}, against log t, is an effective tool for assessing constancy of hazard rate implying exponential distribution.(5)

## Semi-Parametric Survival Analysis Method: Cox-Proportional Hazard Method

The parametric models discussed above specify a specific form for the hazard function over time. As such these models fully characterize the distribution of failure time as a function of time (and covariates). If however, interest is primarily in determining how a new treatment affects survival compared to an old treatment, then a functional form for the hazard over time need not be specified. This leads to a semi-parametric regression models used to describe survival time in a comparative sense, the most commonly adopted of which is the Cox-proportional hazards model.

Cox-proportional hazard model is not a pure parametric method since it does not assume any functional form of distribution of hazard rate. However, it assumes that hazard function of any two individuals is proportional with the ratio being determined by the covariates that is constant over time.(6,7) Clearly, if one is unsure of the functional form of hazard function, adopting a semi-parametric approach would be the preferred alternative to imposing specific parametric assumptions. Considering the semi-parametric assumption, the ratio of hazard functions for any two individuals is constant over time and is determined by the individuals’ covariate values. The latter is nothing but an expression of relative risk.

The proportionality assumption of semi-parametric method can be assessed either graphically by plotting survival curves or by means of a test statistic,(8) dependent on which the null hypothesis is rejected if the *P* value is less than significance level. In graphic representation, proportionality assumption holds true if the two survival curves do not cross each other. Alternatively, complementary log transformation, Log{- Log [S(t)]}, is useful. Plot of the double log of two survival curves i.e. Log{- Log [S(1)]} and Log{- Log [S(2)]}is a straight line contingent to correctness of proportionality assumption.

Besides, the parametric and semi-parametric methods described above, rank regression methods have been used to deal with time to event data in the presence of censoring. Asymptotic normality of estimators is established under certain assumptions. This method makes use of martingale theory and a tightness lemma for stochastic integrals of multi-parameter empirical process.(9)

## Suitability of Survival Analysis Methods

Non-parametric methods i.e. Kaplan Meier product limit method and the Cox-proportional hazards model are the most commonly used methods for survival analysis in public health literature. Kaplan Meier method is suitable when no assumption about the functional distribution of hazard rate with time is made. However, since no assumption is made about hazard rate, no extrapolation of study results beyond the study period is possible. Parametric methods overcome this disadvantage and are used for extrapolation of study results beyond the study period discussed in subsequent section. Parametric and semi-parametric methods also help in ascertaining the influence of covariates on survival times. However, it is inappropriate to assume an exponential distribution universally, especially in case of survival rates following birth and survival rate after a major surgery, contingent upon knowledge that mortality rates are higher in infancy and in immediate post-operative period, respectively, with gradual decline thereafter. Semiparametric methods can be useful in such situations. Cox-proportional method has the additional advantage of being able to ascertain influence of covariates such as demographic factors, nature of diseases and treatment received on hazard/survival rate.

An advantage of Cox-proportional hazard model over the log-rank test following Kaplan Meier method is the ability to use the former with continuous and categorical (after creation of dummy variable) variables.(5) On the other hand the log-rank test cannot deal with continuous variables.

## Extrapolation of Survival Estimates Beyond Study Follow-Up

Epidemiological studies estimating survival are generally conducted for a short follow-up period which is contingent upon recruitment of sufficient number of individuals to provide adequate study endpoints which powers the study to ascertain difference in influence of covariates on time to event at a given significance level. However, despite this relatively shorter followup, it is relevant in many cases to extrapolate results at longer time duration. This is especially pertinent in economic evaluations where time horizon has to be sufficiently large, in many cases life-time horizon, to guide decision-makers for making sound decisions. The above-mentioned statement particularly applies to preventive programs where benefits of a program start appearing and are yielded over longer time duration.

Extrapolation of results requires an assumption of how the present results would vary in course of time i.e. function of hazard rate over time. It may assume that the impact of covariate on hazard rate remains the same after the follow-up period; or it ceases to occur; or an intermediate situation. Alternatively a parametric method may be used to model observed data to extrapolate results. This is an application of Weibull and exponential methods. Lastly, survival curves from other observational studies are matched with present study data. Data from observational study which matches the present survival curve maximally is used to model the results beyond follow-up period.

## Conclusion

We acknowledge that this article does not cover in-depth certain finer points related to methods for survival analysis. Nonetheless, the article can serve as a good note for the beginners who are interested to learn survival analysis. The present essay discusses the role of survival analysis techniques in individual level patient data amidst censoring which have been widely used by health economists, public health professionals, social and behavioral scientists. Right censoring is primarily dealt with by the application of these survival analysis methods, while interval censoring has been dealt with by statisticians using imputation techniques. Left censoring is usually not a problem in thoughtfully designed clinical trials since starting point or beginning of risk period is defined by an event such as randomization or performance of an intervention.

Independence of censoring (or non-informative censoring) is the most important assumption in all methods. However, this assumption may also be null in situations termed as ‘frailty’ i.e. when time between two recurrent events (for instance repeated infections) is the measure of interest, some individuals may have higher chances or an unknown tendency to have recurrent events.(10)

Non-paramteric methods such as Kaplan Meier, Log Rank and Wilcoxon tests are the most commonly used methods for survival analysis. Improved versions with application of prognostic covariate information are being applied which make Kaplan Meier estimates much more robust in the presence of censoring.(11) Impact of covariates affecting the survival rates are commonly dealt with using Cox-proportional hazards method. Recent development of specialized softwares has generated greater interest in application of parametric methods which offer additional advantages and allow extrapolation of results beyond the study follow-up period.
